# Application of Machine Vision Techniques in Low-Cost Devices to Improve Efficiency in Precision Farming

**DOI:** 10.3390/s24030937

**Published:** 2024-01-31

**Authors:** Juan Felipe Jaramillo-Hernández, Vicente Julian, Cedric Marco-Detchart, Jaime Andrés Rincón

**Affiliations:** 1 Valencian Research Institute for Artificial Intelligence, Universitat Politècnica de València (UPV), Camí de Vera s/n, 46022 Valencia, Spain; vjulian@upv.es (V.J.); cedmarde@upv.es (C.M.-D.); 2 Valencian Graduate School and Research Network of Artificial Intelligence (VALGRAI), Universitat Politècnica de València, Camí de Vera s/n, 46022 Valencia, Spain; 3 Departamento de Digitalización, Escuela Politécnica Superior, Universidad de Burgos, 09006 Miranda de Ebro, Spain; jarincon@ubu.es

**Keywords:** computer vision, object detection, depth estimation, precision agriculture

## Abstract

In the context of recent technological advancements driven by distributed work and open-source resources, computer vision stands out as an innovative force, transforming how machines interact with and comprehend the visual world around us. This work conceives, designs, implements, and operates a computer vision and artificial intelligence method for object detection with integrated depth estimation. With applications ranging from autonomous fruit-harvesting systems to phenotyping tasks, the proposed Depth Object Detector (DOD) is trained and evaluated using the Microsoft Common Objects in Context dataset and the MinneApple dataset for object and fruit detection, respectively. The DOD is benchmarked against current state-of-the-art models. The results demonstrate the proposed method’s efficiency for operation on embedded systems, with a favorable balance between accuracy and speed, making it well suited for real-time applications on edge devices in the context of the Internet of things.

## 1. Introduction

In recent years, technologies such as artificial intelligence (AI) [1,2,3], the Internet of things (IoT) [4,5], electronics [6,7,8], and edge computing [9] have become essential for enhancing energy efficiency, autonomy, and sustainability in global agriculture systems. This is especially critical due to the challenges posed by exponential population growth [10], which affects food security and leads to complex sociocultural problems and precarious working conditions in fields in Spain [11].

Harvesting is a fundamental process in the food production chain involving the collection of ripe fruits for consumption or commercial purposes. Technological innovation in this process is pivotal for improving agricultural productivity, soil management, climate resilience, and environmental remediation [12]. Integrating complex data acquisition systems, processing algorithms, and control systems to develop automated harvesting platforms can address these challenges [13,14,15,16,17,18]. Moreover, high-resolution sensors and high-end computers can lead to expensive hardware expenses. However, levering software capabilities such as optimized deep learning algorithms trained with high-resolution data can significantly reduce hardware costs while preserving high-quality data acquisition in new precision agriculture solutions [19,20,21].

One of the crucial tasks in the automation of fruit harvesting is the efficient spatial localization of the fruits. State-of-the-art fruit detection relies on fully convolutional neural networks (CNNs) for an optimal speed-precision balance [22]. Furthermore, integrating depth estimation can strain processing resources, requiring either stereo systems [23,24,25], LIDAR sensors [26], or dedicated monocular networks [27,28,29]. In this sense, this work presents a novel Depth Object Detector (DOD) method: a deep-learning-based lightweight object detection algorithm with monocular depth estimation for cost-effective systems and real-time applications.

The novelty of our approach consists in leveraging the state-of-the-art model You Only Look Once Version 8 (YOLOv8), proposed by Jocher et al. [30]. This involves the integration of a novel regression head designed to model depth estimation as a representative value for each object while concurrently optimizing the network’s computational efficiency. Moreover, due to the absence of a public dataset incorporating fruit detection and depth information, we propose an initial solution by modifying conventional object detection datasets. This requires incorporating representative depth labels for each object using the state-of-the-art monocular depth estimation model MiDaS [31,32]. In future work, we expect to construct an integrated dataset with physical metrics to calibrate and enhance the performance of depth estimation.

The Microsoft Common Objects in Context (COCO) dataset [33] is used to assess the computational performance to validate the proposed architecture size. Regarding fruit detection, the MinneApple dataset [34] is chosen for its uniform images capturing apple orchards at a consistent relative distance from the camera point-of-view. Finally, a quantized version of the proposed method is evaluated on an embedded system, demonstrating its capabilities in terms of size and speed.

The rest of the paper is structured as follows: Section 2 presents the related work; Section 3 describes the proposed DOD method; Section 4 presents and analyzes the different tests against the state of the art; finally, some conclusions are presented in Section 5.

## 2. Related Work

Object detection is a fundamental technique in computer vision that enables computer systems to identify and locate objects within images or videos using advanced algorithms to analyze visual patterns and distinguish objects from the background in a scene. On the other hand, depth estimation is a fundamental technique in computer vision that involves calculating the depth of each pixel in an image. Traditionally, this task has been addressed by using the disparity in stereo or multiview images to provide a basis in pixel coordinates for triangulating the distance of each point from the virtual camera [35,36].

In recent years, thanks to scientific progress and recent attention, neural networks have been able to overcome the depth estimation challenge using datasets composed of monocular images and their depth maps as ground truth, thus representing the multidimensional relationships within the semantic context of the foreground objects and the background of a given scene to infer the depth of each pixel in the image [37,38,39].

Even further, it is possible to optimize the depth estimation by focusing solely on key objects through object detection techniques [24,26,27,28]. This strategy maximizes computational efficiency and optimizes processing time by prioritizing the foreground of the scene, making it an attractive option for real-time applications such as autonomous driving [40,41], robotics [42], surveillance [43], or assisted surgery [44].

### 2.1. Depth Estimation

The current state of the art of monocular depth estimation is defined by Ranft et al. [31,32] (2021, 2022) by their architecture based on vision transformers (ViTs) [45] in place of CNNs as the backbone of dense prediction tasks. The transformer has a global receptive field at a constant and relatively high resolution, allowing for more detailed and globally consistent predictions than fully convolutional networks, especially when a large quantity of training data is available. Therefore, Zhao et al. [46] (2023) present a visual perception architecture that leverages ViTs by taking advantage of the semantic information of a pretrained text-to-image diffusion model in visual perception tasks such as depth estimation.

Further, Peluso et al. [47] (2022) propose an efficient monocular depth estimation method for microcontrollers based on a lightweight CNN with a shallow pyramidal architecture. By using optimization strategies to perform calculations on 8-bit data and mapping the high-level description of the network to low-level layers optimized for the target microcontroller architecture, experimental results show that it is possible to obtain depth estimates sufficiently accurate for objects with large overlap areas.

### 2.2. Object Detection

Among the deep learning architectures that mark the current state of the art of object detection, the open-source algorithm You Only Look Once (YOLO) introduced by Redmon et al. [48] (2015) has stood out for its balance between speed and precision thanks to its evolution through successive iterations that improve previous versions to overcome limitations and improve performance [49]. The YOLOv8 model, proposed by Jocher et al. [30] (2023), establishes the current state of the art in object detection for fully convolutional architectures regarding speed and accuracy.

On the other hand, the revolution of cross-attention models, such as ChatGPT [50], has marked a breakthrough in generative AI for text-to-text, text-to-image, and image-to-image tasks. As an outcome, Meta proposes its open-source SAM (Segment Anything) model by Kirillov et al. [51] (2023) for object detection and semantic segmentation. This architecture consists of a ViT-based image encoder and a cue-guided mask decoder. Chaoning et al. [52] (2023) propose MobileSAM, a more optimal and faster version than SAM, with the same features but fewer parameters, ideal for mobile applications.

### 2.3. Object Detection for Precision Agriculture

Häni et al. [34,53,54] (2019) present MinneApple, a new dataset to advance state-of-the-art fruit detection, segmentation, and counting in orchard environments, providing a large variety of high-resolution images of different apple tree species collected at the University of Minnesota’s Horticultural Research Center (HRC) between June 2015 and September 2016. Additionally, they present a benchmark performance analysis for the tasks using different object detection model architectures based on regions with CNN features (R-CNN) [55] with ResNet50 [56] as the feature extraction backbone, along with their proposed Tiled Faster R-CNN architecture.

Xiang et al. [57] (2021) proposed a system for the detection of loose oil palm fruits using the Faster R-CNN architecture [58] on NVIDIA Jetson TX2 hardware. In their study, 500 images of loose fruits were collected from an oil palm farm in Bukit Bangkong, Selangor, during harvesting. The model achieved an accuracy of approximately 94% for an intersection over union (IoU) threshold equal to 0.5, demonstrating that the developed system was capable of detecting loose oil palm fruits accurately and had the potential to contribute to the development of an automatic fruit-harvesting system.

Nagaraju et al. [59] (2022) proposed a fruit recognition technique based on the  YOLOv5 [60] that detects custard apples, pomegranates, and wax berries. They collected images of fruits in a natural environment and preprocessed them to create a private dataset. With a mean average precision (mAP) of 89.4% at the 0.5 IoU threshold, they demonstrated that their system had significant implications for autonomous fruit-harvesting systems in orchards.

Wu et al. [61] (2023) present a Normal Detection Matched Fruit Counting System (NDMFCS), employing YOLOv4-tiny [62] for object detection, abnormal fruit detection thresholding, and trunk tracking with identity assignment. Results from 10 video sets show significant improvements, with fruit detection precision rising from 89.1% to 93.3%, enhancing overall counting accuracy to 95.0%. NDMFCS demonstrates promise as a technical solution for precise fruit yield estimation in modern apple orchards.

### 2.4. Object Detection with Depth Integration

Wang et al. [27] (2021) presented a real-time object detection and depth estimation approach based on CNNs. For depth estimation, they introduced binocular vision into a monocular-vision-based disparity estimation network and used the epipolar constraint to improve prediction accuracy. Finally, they integrated the 2D location of the detected object with the depth information to achieve real-time depth detection and estimation. The results demonstrated that the proposed approach obtains better results than conventional methods. However, computing was complex and expensive in terms of processing.

Lee et al. [24] (2022) present a simplified approximation of depth in stereoscopic image objects by quantifying depth values into a small number of representative values. This allows for the avoidance of complexity in calculations by estimating only a representative depth value for each object instance and not having to estimate the values of all the pixels that contain the object. Their results on the KITTI dataset [63] demonstrate that despite the low complexity, their approximation method significantly improves object detection performance.

Fan et al. [28] (2022) sought to improve the real-time performance of 3D reconstruction by proposing a novel approach to reduce the consumption of computational resources by extracting significant regions from depth maps by fusing 2D object detection and self-supervised monocular depth estimation.

Usman et al. [26] (2022) introduce a point-pixel fusion system for object detection and classification with depth information for an autonomous driving system. Specifically, they combine the points of a LIDAR sensor with a 2D image, which is processed by an object detection model that extracts regions of interest to determine the depth in the highlighted objects, thus discarding the rest of the LIDAR points and preserving only the regions of interest.

Within the context of this work, the method proposed by Lee et al. [24] for depth estimation is the most relevant to this study, as we propose to address the depth estimation by using a single representative value for each detected object instance on monocular images.

## 3. Proposed Method

**Figure 1:**
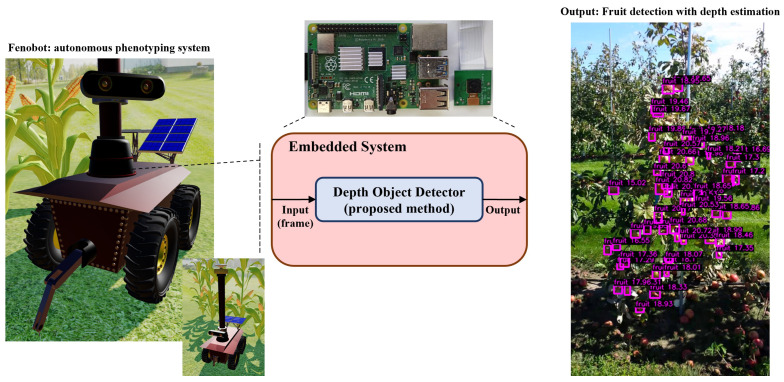
Integration diagram of the DOD method within an autonomous phenotyping robot. The Fenobot images are taken from our simulation. The orchard image is taken from MinneApple.

In precision agriculture, our method is intended to be implemented in autonomous systems for crop harvesting or phenotyping tasks. As described in Figure 1, by the integration with low-cost embedded systems equipped with a digital camera as a photoelectric transducer to obtain 2D RGB images of crops, the output of our proposed method serves as a control signal for various tasks, such as fruit harvesting, fruit counting, disease detection, or other phenotype characteristics, such as the size, width, color, and age of the fruits.

### 3.1. Architecture

**Figure 2:**
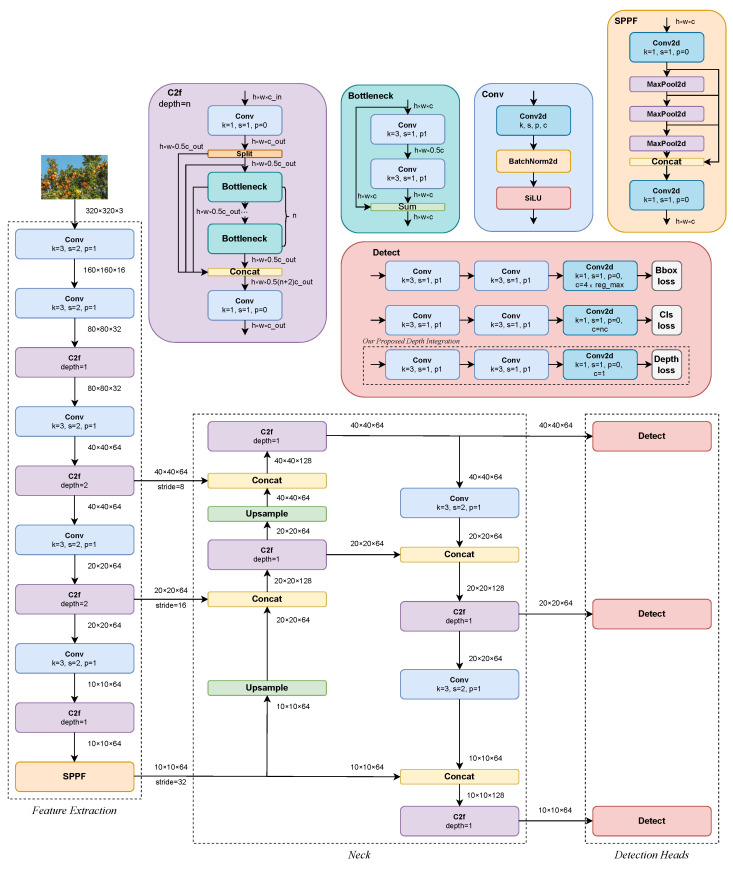
DOD architecture inspired by YOLOv8.

The Depth Object Detector (DOD) architecture is a fully CNN inspired by the object detection architecture of YOLOv8 [30]. It is adapted to reduce the number of parameters for its application on low-cost or edge devices while integrating the depth estimation of each bounding box (bbox) as an extra regression head on the output layers. As shown in Figure 2, the network architecture is mainly composed of the following sections:

Feature extraction: the feature extraction layers are primarily composed of the C2f (Conv-to-Features) block and the SPPF (fast spatial pyramid pooling) [64] block.

Neck: a neck designed as a feedback closed loop, inspired by Efficient Layer Aggregation Networks [65], which enhances the gradient distribution by the shortest and longest gradient path along the network.

Detection heads: the network comprises three output detection layers for different detection scales. Prediction heads represent each one of the following tasks: bbox regression, class classification, and depth value estimation, as our aggregation on the proposed method in this work.

The main blocks that compose the architecture are described as follows:

Conv: Convolution module composed of a 2D spatial convolution defined by a kernel size *k*, a stride size *s*, a padding size *p*, and input and output filter sizes $c_{in}$ and $c_{out}$. The output of the convolution undergoes a 2D batch normalization [66] followed by the SiLU (sigmoid-weighted linear unit [67]) activation function:

(1) $$\mathrm{silu}\left(x\right)=x\left(\frac{1}{1+e^{-x}}\right)$$

Bottleneck: bottleneck block based on ResNet (2016) [56], consisting of two residual-connected convolutional feature extraction modules to mitigate the vanishing gradient problem [68].

C2f: Partial bottleneck block with two convolutional modules between depth-crossing stages *n*. Cross-Stage Partial Networks [69] inspired the C2f, which allows features to be partially preserved, communicated, and combined between different stages of the network. This produces better feature reuse and enables the network to capture more complex patterns and relationships, improving accuracy.

SPPF: A fast spatial pyramid pooling (SPP) [64] module that speeds up computation by pooling features into a fixed-size map. Sequential max-pooling operations aim to separate the most relevant features and significantly increase the receptive field in the context without decreasing the network’s speed.

Detect: detection block composed of three decoupled heads composed of two convolutional modules and a final 2D convolution to predict for each prediction cell:

1. Bounding box regression: The output is an anchor-free [70] distribution of reg_max values for each distance left, right, top, bottom (l,r,t,b) relative to the center of the prediction cell. After linearly projecting the distributions into four-pixel coordinates in the inference process, the width and height of the bboxes are in the range:
  (2) $$x_{1},y_{1},x_{2},y_{2}=\left[0,\;2\left(reg\_max-1\right)max\left(stride\right)\right]$$
  given that:
  (3) $$x_{2}y_{2}-x_{1}y_{1}=rb+lt$$
  The distributional focal loss (DFL) function, proposed by Li et al. [71], introduces the hyperparameter reg_max to prevent the boxes from being too large or too small, ensuring the sensitivity of the predictions. For our proposed DOD method, reg_max=4 and max(stride)=32.
2. Classification: The output is nc logits. For fruit detection, it is only considered one class nc=1. In the case of the COCO dataset [33], the number of classes is nc=80.
3. Depth: The output is one representative depth value as a dimensionless quantity; the closer the object from the foreground, the higher the depth value and vice versa. This quantity is described in detail in Section 3.4.

It is important to note that the training of the network adopts the task-aligned one-stage object detection [72] label assignment strategy to speed up the convergence by selecting a top-*k* number of positive predictions for each ground truth based on a weighted classification and regression score.

### 3.2. Inference Process

**Figure 3:**
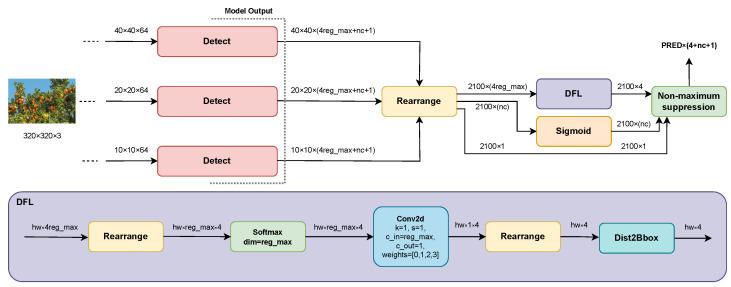
DOD’s inference process.

The detection heads generate a fixed number of predictions for each stride, regardless of the detected objects (see Figure 2). This tensor must be processed by an inference process (see Figure 3) to obtain a filtered result containing only the four-pixel coordinates, the highest confidence class, and the relative depth of each valid object detected in the input image.

The softmax function is applied to obtain a probability vector for each distance distribution. These vectors are then linearly transformed into the four distances through a 2D convolution without gradient with $c_{out}=4$ filters and a kernel size k=1, whose weights are preinitialized as w=[0,1,2,3].

Once the distances *l*, *r*, *t*, and *b* for each prediction are obtained, they are added to the pixel coordinates of the central point of each prediction cell and scaled according to the stride of the level where they were predicted, either 8, 16, or 32. This ultimately yields a bbox for each prediction with coordinates x,y relative to the dimensions of the input image.

Finally, a nonmaximum suppression (NMS) postprocessing technique is used to reduce the number of overlapping bboxes according to the Jaccard index, also known as IoU (see Equation (8)), which measures the degree of similarity between two boxes.

### 3.3. Loss Function

The weights of the network are adjusted by minimizing the mathematical formula described in Equation (4), which is the generalized loss function incorporating individual loss weights and a regularization term with weight decay ϕ. This is achieved using Equation (5) as the weight update rule with a learning rate η and an update velocity term with momentum β, as described in Equation (6). The specialized loss function is described in Equation (7) and is inspired and adapted from Reis et al.’s [73] description.

(4) $$L\left(\theta \right)=\frac{\lambda_{box}}{N_{pos}}L_{box}\left(\theta \right)+\frac{\lambda_{cls}}{N_{pos}}L_{cls}\left(\theta \right)+\frac{\lambda_{dfl}}{N_{pos}}L_{dfl}\left(\theta \right)+\frac{\lambda_{depth}}{N_{pos}}L_{depth}\left(\theta \right)+\phi {∥\theta ∥}_{2}^{2}$$

(5) $$\theta^{t}=\theta^{t-1}-\eta V^{t}$$

(6) $$V^{t}=\beta V^{t-1}+\nabla_{\theta }L\left(\theta^{t-1}\right)$$

(7) $$\begin{array}{c} L=\frac{\lambda_{box}}{N_{pos}}\sum_{x,y}1_{c_{x,y}^{*}}\left[1-q_{x,y}+\frac{{∥b_{x,y}-{\hat{b}}_{x,y}∥}_{2}^{2}}{\rho^{2}}+\alpha_{x,y}v_{x,y}\right]+\frac{\lambda_{cls}}{N_{pos}}\sum_{x,y}\sum_{c\in clases}y_{c}log\left({\hat{y}}_{c}\right)+\left(1-y_{c}\right)log\left(1-{\hat{y}}_{c}\right))\\ +\frac{\lambda_{dfl}}{N_{pos}}\sum_{x,y}1_{c_{x,y}^{*}}\left[-\left(d_{(x,y)+1}-d_{x,y}\right)log\left({\hat{d}}_{x,y}\right)+\left(d_{x,y}-d_{(x,y)-1}\right)log\left({\hat{d}}_{\left(x,y)+1\right)}\right)\right]+\frac{\lambda_{depth}}{N_{pos}}\sum_{x,y}1_{c_{x,y}^{*}}{\left(z_{x,y}-{\hat{z}}_{x,y}\right)}^{2} \end{array}$$

(8) $$q_{x,y}=IoU\left(x,y\right)=\frac{\left|{\hat{\beta }}_{x,y}\cap \beta_{x,y}\right|}{\left|{\hat{\beta }}_{x,y}\cup \beta_{x,y}\right|}$$

(9) $$v_{x,y}=\frac{4}{\pi^{2}}{\left(\arctan\left(\frac{w_{x,y}}{h_{x,y}}\right)-\arctan\left(\frac{{\hat{w}}_{x,y}}{{\hat{h}}_{x,y}}\right)\right)}^{2}$$

(10) $$\alpha_{x,y}=\frac{v}{1-q_{x,y}}$$

(11) $${\hat{y}}_{c}=\sigma \left(\cdot \right)$$

(12) $${\hat{d}}_{x,y}=softmax\left(\cdot \right)$$

where:

- $N_{pos}$ is the total number of cells containing an object (positive predictions).
- $1_{c_{x,y}^{*}}$ is the indicator function for cells with detected objects.
- $q_{x,y}$ is the IoU between predicted and ground-truth bboxes (Equation (8)).
- $\beta_{x,y}$ is a tuple ($x_{coord},y_{coord},width,height$) representing a ground-truth bbox.
- ${\hat{\beta }}_{x,y}$ is a bbox predicted by a respective cell.
- $b_{x,y}$ is a tuple ($x_{coord},y_{coord}$) representing the central point of a ground-truth bbox.
- ${\hat{b}}_{x,y}$ is the central point of a bbox predicted by a respective cell.
- ρ is the diagonal distance of the minimum bbox enclosing both a predicted and a ground-truth bbox.
- $v_{x,y}$ measures consistency in the aspect ratio between predicted and ground truth bboxes based on their width and height, respectively, ($w_{x,y},h_{x,y}$), (${\hat{w}}_{x,y},{\hat{h}}_{x,y}$) (Equation (9)).
- $\alpha_{x,y}$ is a positive compensation where the overlap area factor has higher priority for regression, especially for nonoverlapping cases (Equation (10)).
- $y_{c}$ is the ground-truth label for class *c* for each individual cell, regardless of whether an object is present.
- ${\hat{y}}_{c}$ is the predicted probability for class *c* for each individual cell, regardless of whether an object is present (Equation (11)).
- $d_{(x,y)+1}$ and $d_{(x,y)-1}$ are tuples (l,r,t,b) with values closest to the left and right of a ground-truth bbox whose tuple ($x_{coord},y_{coord},width,height$) has been transformed to a relative distance from the center of a positive prediction cell.
- ${\hat{d}}_{x,y}$ are the probabilities of the predicted 4×reg_max distribution by a cell containing an object.
- $z_{x,y}$ is the representative value of the relative depth to the background scene of the object in the ground-truth bbox.
- ${\hat{z}}_{x,y}$ is the representative value of the relative depth to the background scene of the detected object in the prediction cell.

The first term is the complete intersection over union (CIoU) loss proposed by Zheng et al. [74], which incorporates an improvement over the traditional Jaccard index Loss by considering three crucial geometric factors: the overlapped area, the distance between central points, and the aspect ratio between predicted and reference boxes. It penalizes inaccurate predictions severely.

The second term is the binary cross-entropy (BCE) as the classification loss, allowing each cell to predict one or more classes in the case of multilabel classification. This forces the model to learn the distribution of each class independently.

The third term is the distributional focal loss (DFL) proposed by Li et al. [71], which compels the network to quickly focus on values near the reference box by explicitly increasing the probabilities in the predicted 4×reg_max distribution relative to the values closest (to the left and right) of the reference box.

The fourth term is the mean squared error (MSE), which is the loss for the depth estimation in our proposed method. This loss compels the depth integration as a regression problem for a representative depth value for each object detected.

### 3.4. Depth Integration

The used datasets for evaluation only contain labels for the object detection task. To address the lack of depth labels, we propose the usage of the state-of-the-art ViT model MiDaS, proposed by Ranft et al. [31,32], to predict a representative depth of each label in datasets, as described in Algorithm 1.
**Algorithm 1** Depth extraction.1:Require: object detection dataset *D*, trained model for monocular depth estimation *h*2:Ensure: dataset $D^{\prime }$ with representative depth for each reference object3:**for** each dataset *D* **do**4:    Predict depth map z←h(image)5:    **for** each object **do**6:        Extract object bounding box in depth map $z_{obj}\leftarrow z\cap object$7:        Calculate representative depth box value $z_{obj}^{\prime }\leftarrow 0.5\left(\mathrm{mean}\left(z_{obj}\right)+\max\left(z_{obj}\right)\right)$8:        Store label $obj_{depth}\leftarrow z_{obj}^{\prime }$9:    **end for**10:**end for**

**Figure 4:**
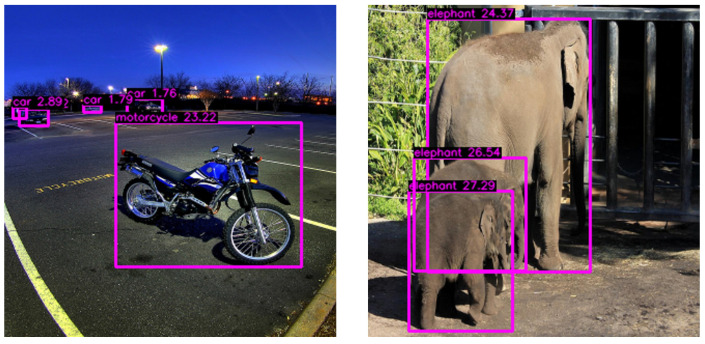
Depth integration into object detection. The higher the representative depth value, the closer the object is to the virtual camera. Images taken from COCO.

This algorithm aims to extract one representative depth value for each object as the mean between the mean and maximum depth intensity within the bounding-box pixels of the depth map synthesized by MiDaS. Moreover, this prediction output does not have a constant range or a physical metric estimation. A higher intensity value in the depth map means the object is closer to the foreground. In contrast, a lower intensity means the object is closer to the background, as seen in Figure 4.

While this approach does not contain a feasible physical quantity for the depth values, it is intended to learn, as a first stage, the occlusion effects, disparity motion, and background segmentation from the detected objects considering its spatial context and semantic information, continuing the line of investigation on monocular depth estimation [37,38,39].

### 3.5. Data Augmentation

**Figure 5:**
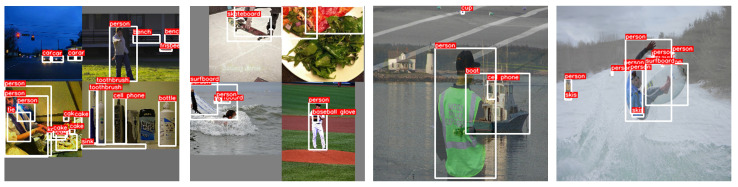
Mosaic augmentation (first two from the left) and MixUp augmentation (last two from the left). Images taken from COCO.

The data augmentation strategies used in training were Mosaic, as introduced in YOLOv8 training by merging four images into one to alleviate batch load and boost spatial context awareness; MixUp, proposed by Zhang et al. [75], which combines two training samples and labels to generate synthetic examples, thus facilitating regularization and improving generalization; ColorJitter, implemented with a 50% probability, which applies color transformations to enhance adaptability to diverse lighting conditions; And lastly, HorizontalFlip, with a 50% probability, which horizontally flips images and labels. Collectively, these techniques aimed to improve model robustness and performance during training. Figure 5 describes some examples of the methods above.

## 4. Results

To evaluate the performance of our DOD method, we conducted the following experimental study:

1. We primarily benchmarked it against the state-of-the-art YOLOv8 model in a common object detection task using the COCO dataset. This direct comparison, conducted under identical conditions, provided insights into the performance disparities due to having nearly three times fewer parameters.
2. Then, we evaluated DOD’s fruit detection performance on the MinneApple dataset and compared the results against the benchmark published by Häni et al. [34,53,54].
3. DOD trained only with MinneApple presented a generalization deficit for relatively large or medium-sized fruits. Therefore, we added some training samples from the Apples dataset [76] and compare the performance disparities on MinneApple.
4. Afterwards, we used MinneApple again to benchmark DOD’s performance with a better generalization on the embedded system, a Raspberry Pi 4 board [77,78], using a 32-bit floating-point precision and a quantized 8-bit signed-integer precision. The quantization process aimed to balance model accuracy and reduced storage for computational requirements, making it suitable for deployment on microcontrollers or embedded systems with limited resources.
5. Finally, an ablation study was performed to analyze the behavior of the different main components of the DOD architecture.

The hardware and libraries used in these evaluations are listed as follows:

- CPU AMD Ryzen 7 5800H 3.20 GHz.
- GPU NVIDIA GeForce RTX 3070 Laptop GPU.
- CPU ARM Cortex-A72 1.5 GHz 64-bits Broadcom SoC BCM2711 on Raspberry Pi 4.
- NVIDIA driver version: 520.61.05.
- CUDA version: 11.8.89.
- PyTorch version: 2.0.1+cu117.
- Torchvision version: 0.15.2+cu117.
- OpenCV version: 4.7.0.
- Albumentation version: 1.3.0.

### 4.1. Common Objects Detection: COCO

**Figure 6:**
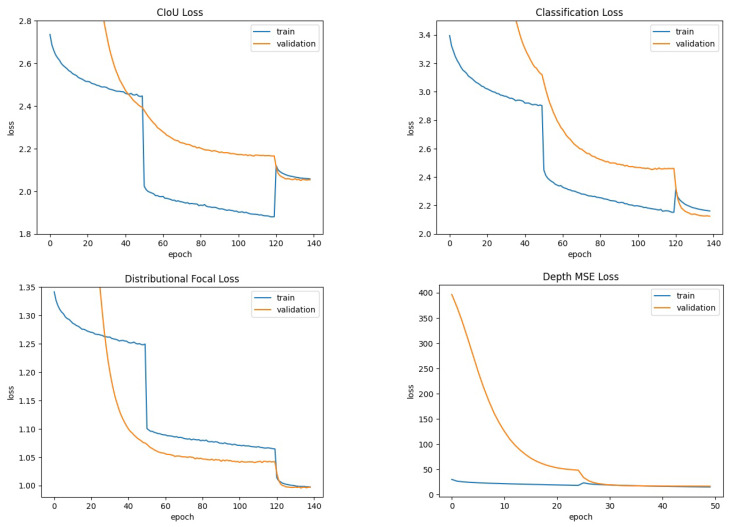
Learning curve for each term of the loss function (see Equation (7)) during the training of DOD on the COCO dataset [33]. Training duration: 55,440 s.

Figure 6 illustrates the learning curve for each term of the loss function (see Equation (7)) during the training of DOD for object detection and depth estimation on COCO. The hyperparameters used in the training strategy were as follows:

- Adam optimizer [79] with its AMSGrad variant [80].
- Three warm-up epochs with a linear learning rate schedule from 0.0001 to 0.001.
- A cosine annealing factor from 0.001 to 0.0001.
- For object detection: 140 training epochs. MixUp: first 50 epochs. Mosaic: first 120 epochs.
- For depth estimation: 50 training epochs. MixUp: none. Mosaic: first 25 epochs.

**Figure 7:**
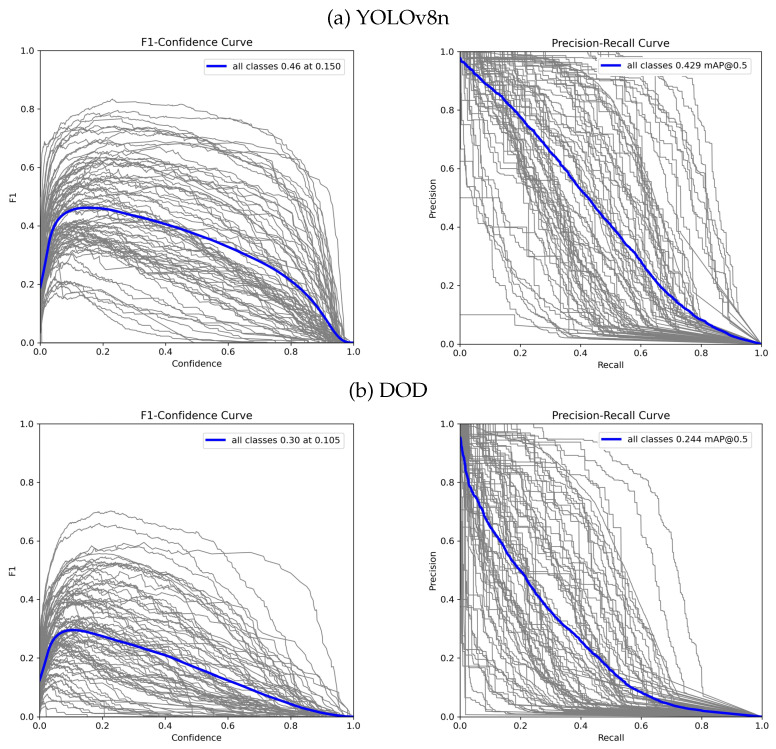
F1–confidence and precision–recall curves for YOLOv8n [30] and DOD evaluated on COCO. F1–confidence shows the harmonic mean of precision and recall for different confidence thresholds for IoU=0.5. Precision–recall illustrates the trade-off between precision and recall for different confidence thresholds for IoU=0.5.

**Figure 8:**
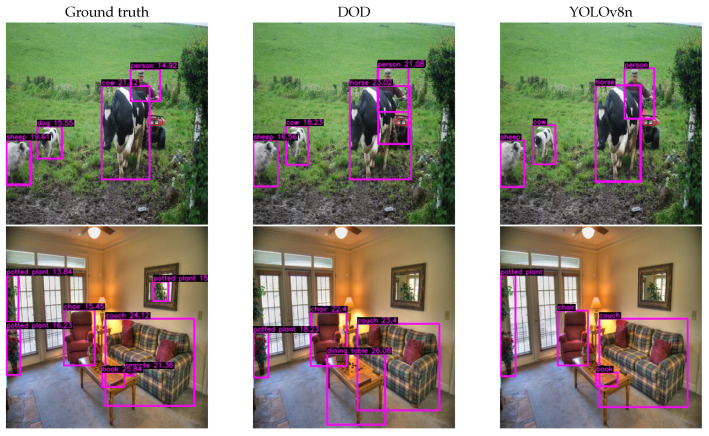
Inference results of DOD and YOLOv8n [30] models after applying nonmaximum suppression with a confidence threshold of 10% (best F1-score, see Figure 7) and an IoU threshold of 60%. The higher the representative depth value, the closer the object is to the virtual camera. Images taken from COCO.

Table 1 presents the results obtained from the evaluation under the same conditions for our proposed DOD method with the lowest validation loss weights found in training and the pretrained YOLOv8n model on COCO. Figure 7 illustrates each detector’s F1–confidence and precision–recall curves. Figure 8 contains some visual results on validation images.

Our proposed one-million-parameter network, trained on 80 classes, shows visually comparable performance to the baseline on the COCO dataset despite the anticipated low scores. While acknowledging a decrease in accuracy, this trade-off is justified by the higher frame rate capability achieved. In edge device scenarios, GPU utilization is crucial for efficient processing. Although the frame rate improvement might not seem significant in GPU-centric evaluations, its impact becomes pronounced in real-world edge device applications, where quicker inference enhances suitability for deployment, striking a balance between accuracy and responsiveness.

### 4.2. Fruit Detection: MinneApple

**Figure 9:**
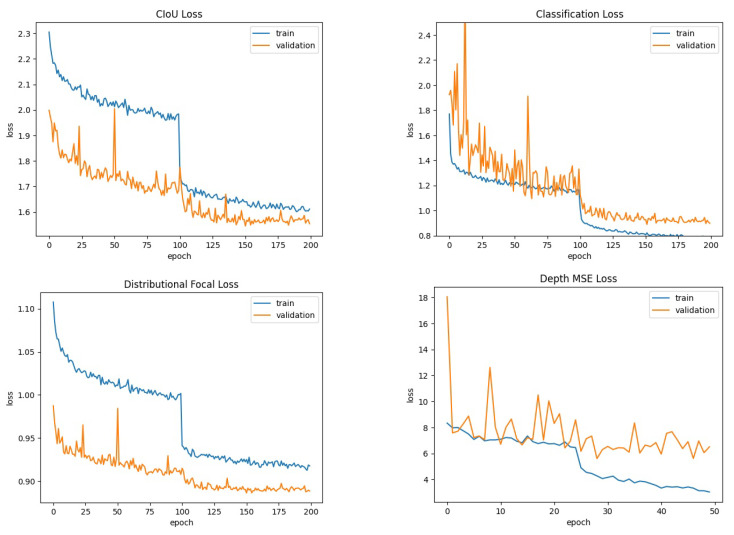
Learning curve for each term of the loss function (see Equation (7)) during the training of DOD on the MinneApple dataset. Training duration: 2426 s.

Figure 9 illustrates the learning curve for each term of the loss function (see Equation (7)) during the training of DOD for object detection and depth estimation on MinneApple. The hyperparameters used in the training strategy were as follows:

- Adam optimizer [79] with its AMSGrad variant [80].
- Three warm-up epochs with a linear learning rate schedule from 0.0001 to 0.001.
- A cosine annealing factor from 0.001 to 0.0001.
- For object detection: 200 training epochs. MixUp: first 100 epochs. Mosaic: not applied.
- For depth estimation: 50 training epochs. MixUp: none. Mosaic: first 25 epochs.

**Figure 10:**
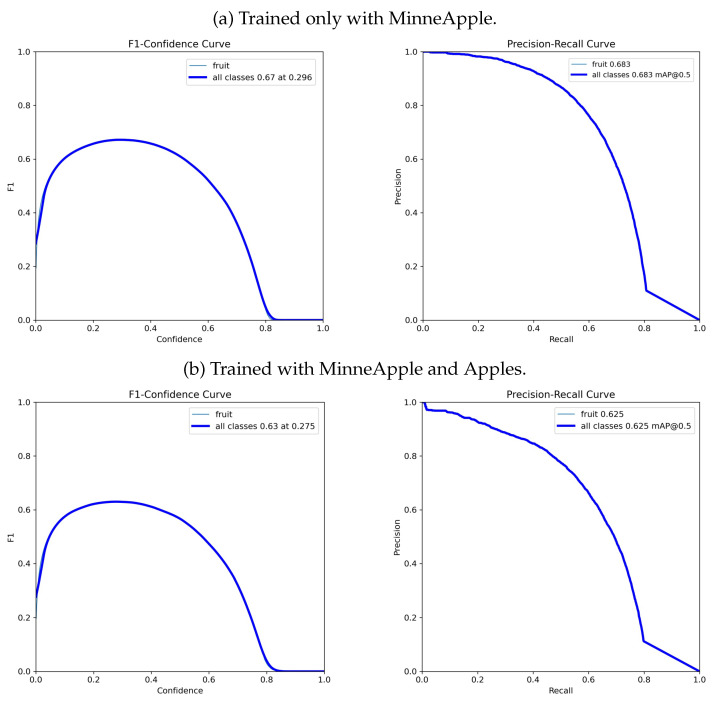
F1–confidence and precision–recall curves for DOD on MinneApple. F1–confidence shows the harmonic mean of precision and recall for different confidence thresholds for IoU=0.5. Precision–recall illustrates the trade-off between precision and recall for different confidence thresholds for IoU=0.5.

**Figure 11:**
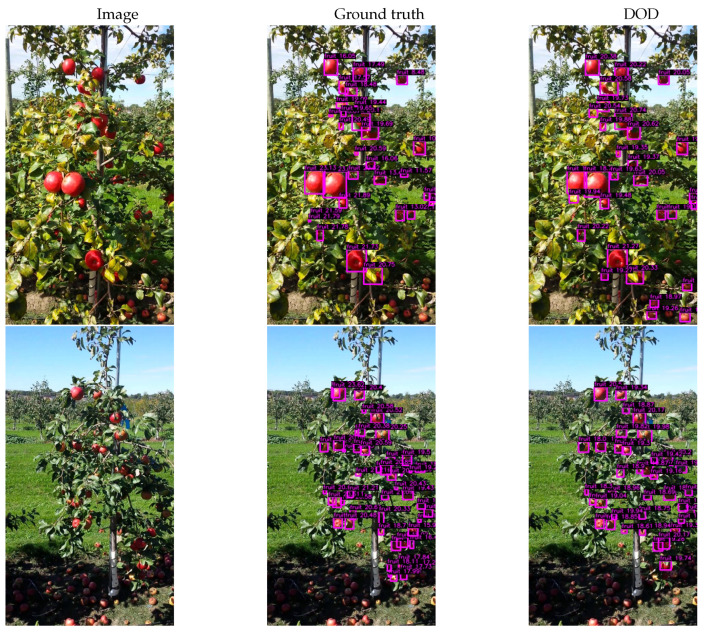
Inference results of DOD trained on MinneApple after applying nonmaximum suppression with a confidence threshold of 27% (best F1-score, see Figure 10) and an IoU threshold of 80%. Images taken from MinneApple.

Table 2 presents the results obtained on MinneApple for DOD with the lowest validation loss weights found in training, along with the results published by Häni et al. [34,53,54] for the following R-CNN-based detection models: Tiled Faster R-CNN [34], Mask R-CNN [81] and Faster R-CNN [58]. Figure 10a illustrates our proposed method’s F1–confidence and precision–recall curves. Figure 11 shows some inference results on validation images.

Furthermore, our proposed model performed well on the MinneApple dataset, achieving notable results with nearly forty times fewer parameters. This underscores the efficiency and effectiveness of incorporating state-of-the-art techniques in the network architecture to obtain remarkable results despite its lean parameter configuration.

#### 4.2.1. Improving Generalization

**Figure 12:**
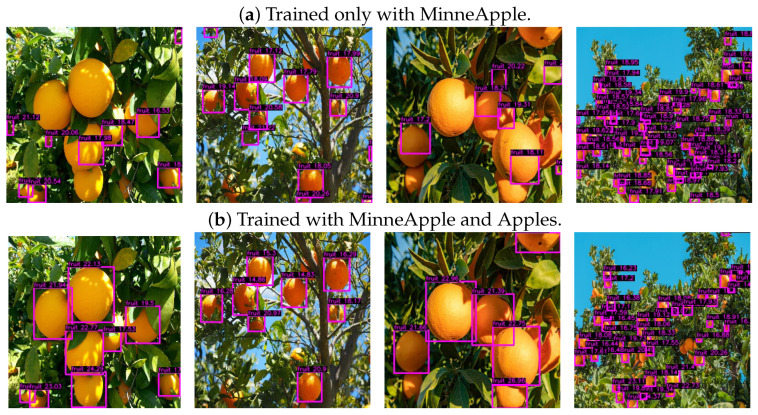
Inference results of DOD versions trained on MinneApple and Apples, respectively, after applying nonmaximum suppression with a confidence threshold of 20% and an IoU threshold of 80%. Images freely accessible from the web.

Figure 12a shows a generalization deficit for relatively large or medium-size fruits when training only with the MinneApple dataset. This is due to the dataset’s homogeneity on its 670 training images, taken in orchard environments, and containing between 1 and 120 object instances per image.

Therefore, adding new training samples with different contexts and sizes can help alleviate this problem. For this reason, we used the Apples [76] dataset with 667 images containing between 1 and 29 apples from different context images taken from the web.

Table 2 presents the results obtained on MinneApple for the improved generalization version of DOD. Figure 10b illustrates its F1–confidence and precision–recall curves. Despite decreasing the detection scores, the better generalization version can now detect much larger fruits, as seen in Figure 12b. Furthermore, the new variety of sizes and spatial contexts in the training samples has improved the spatial awareness of the depth estimation heads, achieving a lower root-mean-square error than the DOD version trained with MinneApple alone.

#### 4.2.2. Quantization

The objective of the quantization process is to optimize the storage space required by the operations and weights of the DOD architecture to operate optimally on microcontrollers or embedded systems (see Figure 1). The methodology used in the quantization process is described as follows:

1. Initialize the DOD with the weights of the best-trained version.
2. Create a quantizable version of the DOD by specifying the operations to be quantized using Pytorch’s Quant/DeQuant placement methods [82]. The library only supports quantizing the following operations: 2D convolution, batch normalization, linear layer, and rectified linear unit (ReLU) activation. The architecture proposed in this work (see Figure 2) uses the sigmoid linear unit (SiLU) activation function for its superior performance in the state of the art compared to ReLU. Therefore, the only quantizable operations in the proposed model are 2D convolutions and their batch normalization.
3. Copy the weights of all operations from the DOD in 32-bit floating-point precision to the quantizable model.
4. Apply the QNNPACK (Quantized Neural Networks PACKage) [83] quantization method integrated into Pytorch [82] to convert from 32-bit floating-point precision to 8-bit signed-integer precision.
5. Calibrate the quantized model using a small number of inference steps on the validation dataset. This is performed to identify the operating ranges of quantized operations and assign the most optimal variable type for storing each weight and operation.

**Figure 13:**
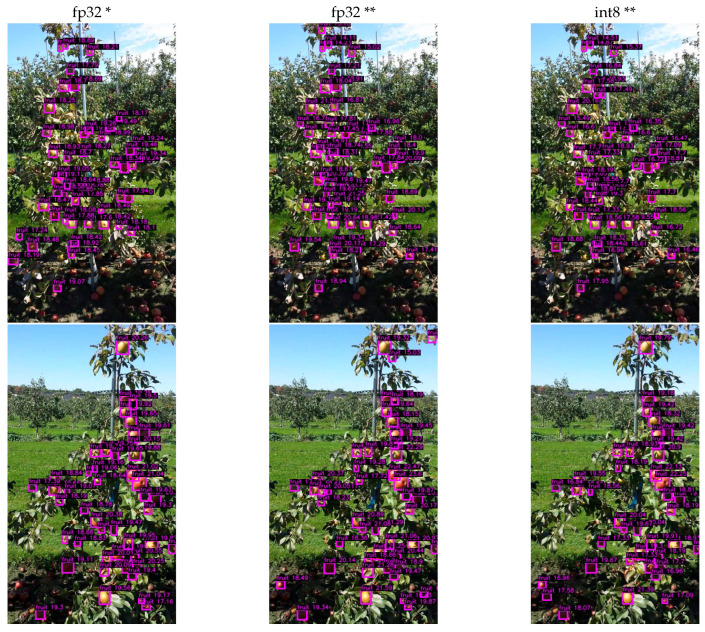
Inference results of different precision versions of the DOD method trained with MinneApple and Apples [76] datasets, respectively. Nonmaximum suppression with a confidence threshold of 20% and an IoU threshold of 80%. Images taken from MinneApple. * Trained only with MinneApple. ** Trained with MinneApple and Apples.

Table 3 lists the results obtained on MinneApple for the improved generalization version of DOD, using 32-bit floating-point precision and 8-bit signed-integer precision. See Figure 13 for a visual comparison.

Although a few of the scores decrease, the int8 quantized version gains a slight frame rate increase on high-end devices. On the other hand, for low-end devices such as the Raspberry Pi 4, kernel implementations of the quantized operations have yet to be optimized to show a noticeable impact on frame rate capabilities [84]. Moreover, the memory footprint reduction is almost three times that of the fp32 version, thus impacting low-end devices.

### 4.3. Ablation Study

Inspired by the ablation study of Meyes et al. [85] for VGG-19 [86], we conducted ablations of groups of similar filters with proportions of 10%, 25%, and 50% relative to the total number of filters in each Conv, C2f, and Detect blocks in the network (see Figure 2). Filter similarity within a group was based on the absolute Euclidean distance of the normalized filter weights. Ablations were performed by manually setting the weights and biases of all incoming connections for a filter to zero, effectively nullifying any activation from that filter. The effect of ablations was evaluated by testing the detection and depth estimation performance of the network on MinneApple.

**Figure 14:**
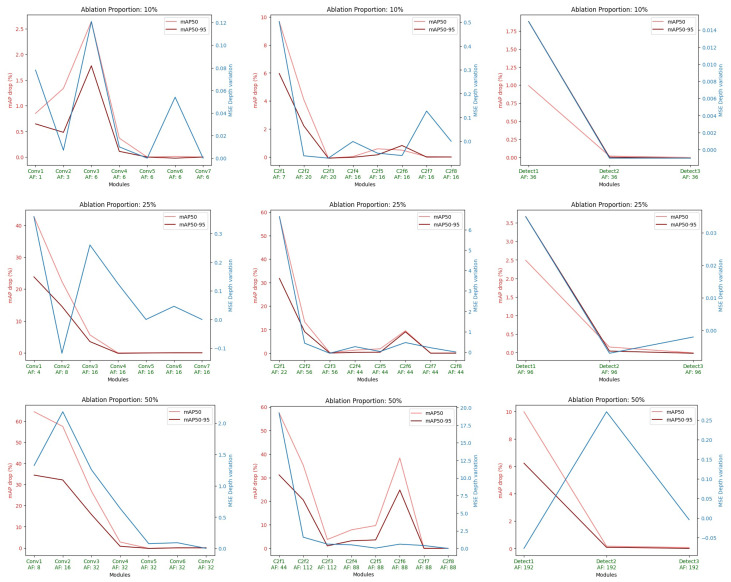
Effect on the evaluation metrics of ablations of different amounts (first row: 10% of layer filters; second row: 25% of layer filters; third row: 50% of layer filters) in all convolutional layers of the blocks (left: Conv block; center: C2f block; right: Detect block) that compose the DOD architecture (see Figure 3). AF is for “Ablated Filters”.

Figure 14 shows the results obtained in the ablation study of the DOD method with 32-bit floating-point precision and trained only in MinneApple. The results of the ablation with proportions of 10%, 25%, and 50% suggest that the initial blocks of the architecture (i.e., Conv1, Conv2, C2f1, Conv3, C2f2) play a crucial role in the information on which the network relies for fruit detection and depth estimation. This can be interpreted from the perspective that these blocks lay the foundations for the connections in the neck of the network, with Conv1 and C2f1 being particularly significant since their ablation represents the maximum performance loss. In the ablation with a proportion of 10%, it is possible to infer that Conv3 contains essential information for communication between the network’s neck and the input image.

On the other hand, from the overall ablation study, it can be deduced that the other blocks in the architecture (i.e., Conv4, C2f3, Conv5, C2f4, Conv6, C2f7, Conv7, C2f8), excluding C2f5 and C2f6, do not contribute any information to the network. However, this behavior is expected when considering the discussion in Section 4.2.1 regarding the nature of MinneApple images. When detecting such small fruits within an image, activation will only occur at the first prediction level (i.e., 40x40x64 features), responsible for detecting smaller objects in an input image due to its smaller stride. This argument is supported by the 50% proportion ablation, where C2f6, fed back by C2f5 and feeding Detect 1, contains crucial information for MinneApple fruit detection. Moreover, among the three blocks in the Detect module, Detect 1 is the only one impacting the network’s performance, emphasizing the behavior described before and the determining role of the neck designed as a feedback closed loop, such as the impact of Efficient Layer Aggregation Networks [65] on current state-of-the-art deep learning architectures.

## 5. Conclusions and Future Work

This work presents the Depth Object Detector (DOD) method as a novel computer vision method for object detection with depth estimation for real-time applications in low-cost embedded or microcontroller systems. The current state of the art in object detection inspired the proposed method’s conception, design, implementation, and operation.

The detection capability of the proposed model was validated through an evaluation on the COCO dataset [33] and a comparison with the YOLOv8 model, which sets the current state of the art. Despite obtaining lower metrics, the proposed method achieved satisfactory visual results in this complex task with 80 classes, all with an architecture of approximately 1 million parameters.

On the other hand, performance in fruit detection was evaluated on the MinneApple dataset [34]. The results exceeded expectations by achieving higher metrics than the method proposed by Häni et al. [53,54], with at least 40 million parameters. The visual results and metrics validated the effectiveness and accuracy of DOD for this task.

Regarding depth estimation, the evaluation of the proposed method was limited to the MSE due to the lack of an analogous method to that evaluated in these tasks as well as datasets for detection with depth labels obtained through reliable physical measurements, at the time of the publication of this work.

In summary, the main contribution of our proposed method lies in integrating depth estimation as a regression head inside a lightweight object detection architecture. By using MiDaS to predict depth labels for conventional object detector datasets, the network can learn to identify occlusion effects and semantic background segmentation in parallel with object detection. This work marks a path for research into integrating these two techniques in monocular vision systems with low-cost hardware and efficient deep learning architectures.

In future work, it will be necessary to adjust the depth values obtained in detection by calibrating the model with measurements from physical instruments. This process will allow us to collect and analyze many experiments, which, in turn, can be used to retrain and adjust the accuracy in the depth estimation of the DOD.

Finally, the results indicate that the quantized DOD method is well suited for deployment on resource-constrained embedded systems, such as the Raspberry Pi. The reduced storage requirements and the efficient inference speed make it a viable solution for real-time applications with low-cost hardware for deployment in various applications that demand lightweight and efficient object detection with depth estimation. Moreover, the ablation study of the proposed method suggests that in future work, it will be possible to reduce large portions of the architecture network without compromising performance, as long as the nature and homogeneity of the target input images are considered.

The source code is available for reproducibility purposes at the GitHub repository: https://github.com/Jaramilloh/Depth-Object-Detector-DOD, accessed on 16 January 2024.

## Figures and Tables

**Table 1:** Evaluation metrics obtained by our DOD proposed method and the state-of-the-art YOLOv8 trained and evaluated on COCO. P (%): precision for the best confidence threshold. R (%): recall for the best confidence threshold. mAP 50 (%): mean average precision for IoU=0.5. mAP 50–95 (%): mean average precision for IoU∈[0.5,0.95;0.05]. MSE depth: mean squared error of depth estimation. Vel. (fps): average inference time for four times the validation partition with a batch of unit size. Parameters (M): number of parameters. Size (MB): storage memory size.

Model	P (%)	R (%)	mAP 50 (%)	mAP 50–95 (%)	MSE Depth	Vel. CPU * (fps)	Vel. GPU * (fps)	Parameters (M)	Size (MB)
YOLOv8n	59.3	39.7	42.8	29.3	-	47.3	83.8	3.15	6.23
DOD	41.3	25.9	24.3	12.3	59.3	57.1	84.7	1.06	4.24

* CPU: AMD Ryzen 7 5800H 3.20 GHz. GPU: NVIDIA GeForce RTX 3070 Laptop GPU.

**Table 2:** Evaluation metrics obtained on MinneApple by the proposed DOD method and the different architectures benchmark by Häni et al. [34,53,54]. P (%): precision for the best confidence threshold. R (%): recall for the best confidence threshold. mAP 50 (%): mean average precision for IoU=0.5. mAP 50–95 (%): mean average precision for IoU∈[0.5,0.95;0.05]. MSE depth: mean squared error of depth estimation. Parameters (M): number of parameters.

Model	P (%)	R (%)	mAP 50 (%)	mAP 50–95 (%)	MSE Depth	Parameters (M)
DOD *	73.7	60.8	68.5	35.7	9.4	1.1
DOD **	68.6	58.1	62.4	31.9	8.5	1.1
TF-RCNN	-	-	63.9	34.1	-	≈41
F-RCNN	-	-	77.5	43.8	-	≈41
M-RCNN	-	-	76.3	43.4	-	≈63

* Trained only with MinneApple. ** Trained with MinneApple and Apples.

**Table 3:** Evaluation metrics obtained by DOD evaluated on MinneApple. P (%): precision for the best confidence threshold. R (%): recall for the best confidence threshold. mAP 50 (%): mean average precision for IoU=0.5. mAP 50–95 (%): mean average precision for IoU∈[0.5,0.95;0.05]. MSE depth: mean squared error of depth estimation. Vel. (fps): average inference time for four times the validation partition with a batch of unit size. Parameters (M): number of parameters. Size (MB): storage memory size.

Model	P (%)	R (%)	mAP 50 (%)	mAP 50–95 (%)	MSE Depth	Vel. AMD * (fps)	Vel. ARM ** (fps)	Parameters (M)	Size (MB)
DOD (fp32)	68.6	58.1	62.4	31.9	8.5	27.2	2.14	1.04	4.18
DOD (int8)	67.2	57.8	61.4	30.4	9.3	33.6	2.34	1.04	1.32

* CPU: AMD Ryzen 7 5800H 3.20 GHz. ** CPU: ARM Cortex-A72 1.5 GHz 64-bits Broadcom SoC BCM2711 on Raspberry Pi 4.

## Data Availability

Data are contained within the article.
